# Effects of Lutein and Astaxanthin Intake on the Improvement of Cognitive Functions among Healthy Adults: A Systematic Review of Randomized Controlled Trials

**DOI:** 10.3390/nu12030617

**Published:** 2020-02-27

**Authors:** Rui Nouchi, Takahiko Suiko, Eriko Kimura, Hiroki Takenaka, Michiaki Murakoshi, Akira Uchiyama, Megumi Aono, Ryuta Kawashima

**Affiliations:** 1 Department of Cognitive Health Science, Institute of Development, Aging and Cancer (IDAC), Tohoku University, Sendai 980-8575, Japan; 2 Smart Aging Research Center (S.A.R.C.), Tohoku University, Seiryo-machi 4-1, Sendai 980-8575, Japan; ryuta@tohoku.ac.jp; 3 Research and Development Headquarters, Lion Corporation, Edogawa, Tokyo 132-0035, Japan; t-suiko@lion.co.jp (T.S.); eriko-f@lion.co.jp (E.K.); takehiro@lion.co.jp (H.T.); michim@lion.co.jp (M.M.); akruchym@lion.co.jp (A.U.); meg-y@lion.co.jp (M.A.); 4 Department of Functional Brain Imaging, Institute of Development, Aging and Cancer (IDAC), Tohoku University, Sendai 980-8575, Japan

**Keywords:** lutein, carotenoids, astaxanthin, intervention, episodic memory, inhibition

## Abstract

Background: Fruits and vegetables are generally rich in antioxidants such as carotenoids. Consumption of carotenoids is expected to have benefits on cognitive functions in humans. However, previous randomized controlled trials (RCT) using carotenoids have reported inconsistent results. Therefore, this systematic review (SR) aimed to summarize the effect of carotenoid intake on cognitive functions in humans. Method: PubMed, Cochrane Library, Web of Science, and PsychoINFO were searched for research papers on carotenoid intake with the criteria that 1) oral carotenoid intake was evaluated using RCTs, 2) participants were healthy young, middle-aged, or older, and 3) cognitive functions were measured using RCTs. Results: Five studies using lutein and two studies using astaxanthin met the inclusion criteria. Consumption of lutein and its isomer showed consistent results in selective improvement of visual episodic memory in young and middle-aged adults while inhibition was observed in middle-aged and older adults. One of the two included astaxanthin studies reported a significant improvement of verbal episodic memory performance in middle-aged adults. Conclusion: This SR showed that the 10 mg lutein per day for twelve months can lead to improvement of cognitive functions. Due to the small number of studies, it is difficult to conclude whether astaxanthin would have a positive effect on cognitive functions.

## 1. Introduction

Cognitive functions are mental processes that include memory, processing speed, executive function, and attention. Cognitive functions change with age. The peak of cognitive function is around twenty or thirty years of age, and then cognitive functions decline after fifty or sixty years of age [1]. Cognitive functions are affected by dietary habits and nutrition consumption [2]. For example, eating breakfast affects cognitive functions in children, young adults, and older adults [3,4]. Mediterranean diets have positive effects on cognitive functions [5]. Many cross-sectional and longitudinal cohort studies have revealed that consumption of fruits and vegetables is positively associated with cognitive functions [6,7]. Fruits and vegetables are generally rich in antioxidants such as flavonoids and carotenoids, which are associated with higher cognitive functions [8] and a lower risk of dementia [9] when included in diets. Nutritional intervention studies have demonstrated that intake of flavonoids and carotenoids improve cognitive functions [10,11]. Although at least two systematic reviews (SR) and meta-analyses have concluded that flavonoids benefit cognitive health [12,13], no SR has investigated the benefits of carotenoids on cognition, therefore necessitating this SR.

Carotenoids are a widely distributed group of naturally occurring pigments, usually red, orange or yellow in color, of which over 750 compounds have been identified [14]. Two chemical classes of carotenoids have been established; the hydrocarbon-based carotenes and the xanthophylls, which also contain oxygen apart from the hydrocarbon component. Generally, carotenoids are one of the derivatives of tetraterpenoids, having a basic skeleton containing C_40_H_56_ and produced from eight isoprenoid units. Because of their characteristically long polyene structure comprising 9 to 11 conjugated double bonds, carotenoids demonstrate strong antioxidant effects on singlet oxygen, which is one of the reactive oxygen species (ROS) [15], as well as protective effects from optical damage through absorption of blue to green light energy [16]. Humans cannot synthesize carotenoids but need them for various functions [17], therefore human levels of carotenoids mainly come from green leafy vegetables.

Meta-analyses and SRs have suggested an association between carotenoids and a reduced risk of several chronic health disorders including some forms of cancer, heart disease, and eye degeneration [18]. In addition, narrative or hand reviews have indicated that the intake of carotenoids affects cognitive functions [19]. Notably, recent intervention studies using randomized controlled trials (RCTs) have demonstrated a positive effect of the intake of carotenoids on cognitive functions [20,21]. However, to the best of our knowledge, there is no SR of RCTs to assess the benefits of carotenoids on cognitive function. Therefore, we aimed to review the scientific evidence related to the beneficial effects of carotenoid on cognition.

## 2. Materials and Methods 

### 2.1. The SR Protocol and Registration

The SR protocol was designed using the International Prospective Register of Systematic Reviews (PROSPERO) with the registration number CRD42018110984 (https://www.crd.york.ac.uk/prospero/display_record.php?RecordID=110984). The protocol followed the statement and general principles of Preferred Reporting Items for Systematic Reviews and Meta-Analyses (PRISMA)[22] (Supplementary Table S1).

### 2.2. Search Strategy

Our review question was “Do xanthophyll and carotene carotenoids improve cognitive functions in healthy adults?” To that effect, the electronic databases PubMed, the Cochrane Library, Web of Science, and PsycINFO were searched using specific search terms (Supplementary Table S2). Databases within the Cochrane Library included the Cochrane Database of Systematic Reviews, the Cochrane Central Register of Controlled Trials, the Cochrane Methodology Register, the Database of Abstracts of Reviews of Effects, the Health Technology Assessment Database, and the National Health Service Economic Evaluation Database. The search strategy comprised of terms related to or describing the intervention, and the search terms were adapted based on database-specific filters of each bibliographic database where available. Only English language articles were included and their publication period was unrestricted.

### 2.3. Detail of Included Studies

#### 2.3.1. Types of Study

Only RCTs assessing the benefits of carotenoid intake were included.

#### 2.3.2. Participants/Population

Inclusion in the SR was reserved to studies where the trial participants were men and women aged 18 years or older. For trials that included a mix of individuals older and younger than 18 years, a study was included if at least 90% of its trial participants were aged 18 years or older at baseline, or if the outcomes for the two age groups could be separated. This SR only included studies of individuals without cognitive impairment. Within any set of participants that fit the inclusion criteria, participants with a current diagnosis or history of Alzheimer’s disease, dementia, stroke, head injury, depression, or other neurologic disorders were excluded.

#### 2.3.3. Intervention and Control

Only studies where the intake of carotenoids was oral were included in the review. Participants also had to have taken only one type of carotenoid, however, the simultaneous intake of two or more types of carotenoids was allowed if the combined carotenoids were of similar function and biodistribution (e.g., lutein and zeaxanthin) [23]. Studies were only admitted to the SR if two or more groups of carotenoids were administered and an orally taken placebo was included in the treatment design.

### 2.4. Main Outcomes

The following indicators of cognitive function were considered based on availability of a validated measurement technique: Overall cognitive function, memory, executive function, attention, verbal fluency, and reasoning. Computerized validated measurement techniques used included the Alzheimer’s Disease Assessment Scale cognitive subscale (ADAS-cog), Mini-Mental State Examination (MMSE), Repeatable Battery for the Assessment of Neuropsychological Status (RBANS), Cambridge Cognition Examination (CAMCOG), the Cambridge Neuropsychological Test Automated Battery (CANTAB), and the Central Nervous System Vital Signs test battery (CNSVS).

### 2.5. Data Extraction (Selection and Coding)

The titles or abstracts of studies were retrieved using the SR’s search strategy. Two reviewers independently screened any studies from additional sources for their fulfilment of the inclusion criteria. Full study reports of all potentially eligible studies were then retrieved and two reviewers independently assessed them for eligibility. Any disagreements between the two reviewers over the eligibility of particular studies were resolved through discussion [24].

### 2.6. Risk of Bias Assessment

Two reviewers independently evaluated the risk of bias in the included studies by using the modified Delphi list [25]. This list was designed based on the Delphi list [26]. To enhance the quality assessment, additional considerations included: Details of random allocation methods, an adequate description of the control/comparison group, between-group statistical comparisons, reports of dropouts, and reports of the consolidated standards of the reporting trials’ (CONSORT) statement [27]. Disagreements between the reviewers on the risk of bias in particular studies were resolved by discussion [24].

### 2.7. Data Synthesis

A qualitative synthesis was carried out to summarize each ingredient’s effectiveness in order to account for the heterogeneity of outcome assessments. We calculated the effect size as the standardized mean difference (SMD), which is equal to Hedge’s g, of the pre-intervention and post-intervention cognitive test score by using Review Manager 5.3 (Cochrane Collaboration, Copenhagen, Denmark).

## 3. Results

### 3.1. Results of the Search

**Figure 1:**
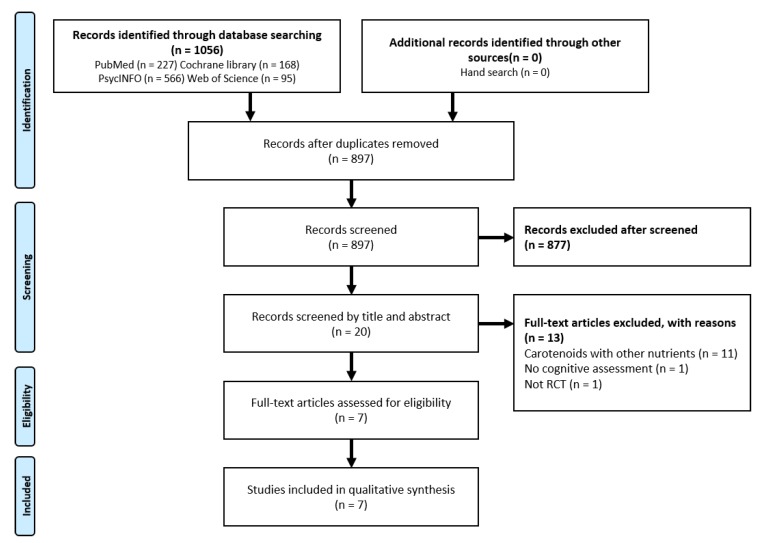
Study selection flow chart.

The electronic database searches performed between November and December 2018 retrieved 1095 abstracts. After deduplication and screening titles or abstracts, 20 potentially eligible studies were identified. Among the 20 studies, 13 were excluded for not having groups treated with carotenoids alone (*n* = 11), having no measurement of cognitive function (*n* = 1), or no RCT design (*n* = 1). The study selection process is presented in the PRISMA flow chart (Figure 1).

### 3.2. Included Studies

Seven trials fulfilled the inclusion criteria of this systematic review, and all included studies had parallel designs (Table 1). Two studies [20,28] were performed as sub-studies of clinical trials that investigated the effects of macula carotenoid intake for improvement in visual function in a normal population and prevention of age-related macular degeneration.

### 3.3. Participants

The sample sizes ranged from 44 to 91. The mean age of participants ranged from 21.21 to 73.74 years. Among the seven studies, one study included young college students [29], three studies included middle-aged adults (aged 40–60 years) [20,30,31], and three studies included elderly individuals (aged over 60 years) [21,28,32]. Four of the trials were conducted in the United States [21,28,29,32], two in Japan [30,31], and one was conducted in Ireland [20].

All trials were restricted to cognitively healthy individuals and all participants were free of retinal disease. One study reported that the amount of Macular Pigment (MP) at baseline was low (i.e., MP at 0.23° of eccentricity ≤0.55 optical density units). MP can directly reflect the function of eye protection from high energy lights and correlate with concentrations of macular carotenoids in the brain [33].

### 3.4. Intervention

Intervention durations of the included studies were as follows: Four studies were conducted for 1 year [20,21,29,32], one study was 4 months long [28], one study was conducted for 12 weeks [30] and one study was conducted for 8 weeks [31].

In five studies [20,21,28,29,32], participants received lutein and its isomer in various capsulated amounts. One study reported that participants consumed a capsule containing 10 mg lutein, 2 mg zeaxanthin, and 10 mg meso-zeaxanthin with a meal [20]. Three studies reported that participants consumed a capsule containing 10 mg lutein and 2 mg zeaxanthin with the highest fat meal [21,29,32]. One study reported that participants consumed a capsule containing 12 mg lutein with a fatty energy drink [28].

In two studies [30,31], participants received pills or jellies containing various amounts of astaxanthin. One such study reported that participants consumed pills containing 6 mg or 12 mg of astaxanthin after breakfast [24], The other study reported that participants consumed jellies containing 8 mg of astaxanthin after breakfast and dinner [25].

### 3.5. Outcome

The outcome measures employed by all the studies were as shown in Table 2, and the results of their qualitative synthesis were as described in Table 3. Overall, 80 cognitive test outcomes were used. In this SR, the cognitive tests were divided into seven cognitive domains (verbal and visual episodic memory, short-term memory/working memory, reasoning, attention, inhibition, shifting, processing speed). Serum lutein (SL), which is correlated with cognitive function [34], and MP were measured. Lutein trials [20,21,28,29,32] and astaxanthin trials [30,31] were not integrated because lutein (and its isomers) and astaxanthin showed different pharmacokinetics [35]. Lutein trials using lutein and its isomers were integrated and discussed because the lutein and its isomers showed similar in vivo distribution and biological function [36]. Published per-protocol data [21,28,29,30,31,32] and unpublished data were used as received from Dr. Power [20] for conducting qualitative synthesis.

### 3.6. Lutein (and its isomers) Intervention

#### 3.6.1. Macular Pigment (MP)

Four studies measured MP [20,21,29,32], with three measuring it as macular pigment optical density (MPOD) using customized heterochromatic photometry (cHFP) [21,29,32]. The mean of MPOD after the intervention was statistically higher than before intervention (MPOD change shows +0.07~0.09) and improved compared to the control group. The remaining study measured MP volume using dual-wavelength autofluorescence (AF) which operates based on different principles to cHFP [20]. MP volume is the sum of MPOD. MPOD is dimensionless. Therefore, MP volume is also unitless [37]. Like in the other studies, MP volume after the intervention was also higher than before (MP volume change shows +2558).

#### 3.6.2. Serum Lutein (SL)

Four studies [20,21,28,29] measured SL, mostly using the same method. The SL was statistically improved compared to the control group (active: +0.475~0.773 umol/L, control: −0.018~+0.158 umol/L). When middle-aged and senior participants took lutein and zeaxanthin, there was a trend of increasing SL (change SL: 0.773~0.647 umol/L) compared to younger people (change SL: 0.475 umol/L).

#### 3.6.3. Episodic Memory (Visual Stimuli)

Seven episodic memory (visual stimuli) outcomes were measured in four studies (Table 3) [20,21,28,29]. using; CANTAB [38]^,^ PAL, CNSVS [39], VIM, and the MIR apartment test [40]. Statistically significant correlations between SL concentration and amount of MP were reported in only PAL (errors adjusted for stage 6) (SL: *r* = −0.346, *p* = 0.006, MP: *r* = −0.342, *p* = 0.005). The tests of episodic memory (visual stimuli) performed for younger and middle-aged individuals (PAL memory score, PAL errors adjusted for stage 6, visual memory of CNSVS) [20,29] indicated statistically significant improvement, but not in older individuals [21,28]. However, the SMD of the post-intervention (effect size) in all studies was small (SMD < 0.5) or could not be calculated because the score of the post-intervention was not reported.

#### 3.6.4. Episodic Memory (verbal stimuli)

Twenty-one Episodic Memory (verbal stimuli) outcomes were measured in five studies [20,21,28,29,32] (Table 3) using CANTAB VRM, Wechsler memory scale paired associates learning test [41], CNSVS VEM, Shopping List Task [42], and Word List Memory Test [43]. Only the Number of Trials to learn the list of Shopping List Task indicated statistically significant correlation with SL (correlation coefficient indicated 0.30, *p* < 0.05). VRM-delayed intrusion errors indicated statistically significant correlation with MP (*r* = 0.306, *p* = 0.033). Only VRM-delayed intrusion errors [20] showed statistically significant improvement, and the SMD of the outcome was small.

#### 3.6.5. Short-term Memory/Working Memory

Four short-term memory/working memory outcomes were measured in one study [28] (Table 3) using forward and backward digit span [44]. No statistically significant correlations were reported with SL and there was no statistically significant improvement in the forward and backward digit span; the SMD of the outcome was also small.

#### 3.6.6. Reasoning

Two reasoning outcomes were measured in two studies [21,29] (Table 3) using the CNSVS NVRT. The correlation between NVRT score and MP in older adults was statistically significant (*r* = 0.45, *p* = 0.04) [21], but there was no report of the correlations in younger adults [29]. Correlations with SL were not reported in the two studies. No intervention effects were observed in older adults [21], however, a sub-analysis in younger adults showed that the score change of NVRT statistically improved in MP increasers compared with participants in which no change was observed [29]. The SMD of the post-intervention could not be calculated because the score of the post-intervention was not reported.

#### 3.6.7. Attention

Two Complex attention outcomes were measured in two studies [21,29] (Table 3) using CNSVS ST & SAT, and CPT. The correlation between the complex attention score of the CNSVS and MP in older adults allocated to the intervention group was statistically significant (*r* = −0.31, *p* = 0.04) [21], but the correlation was not reported in younger adults [29]. Correlation with SL was not reported in two studies. Additionally, intervention effects were observed in older adults [21], and a sub-analysis detected an improvement in young MP increasers [29]. The SMD of the post-intervention also could not be calculated because the score of the post-intervention was not reported.

#### 3.6.8. Inhibition 

Seven Inhibition outcomes were measured in four studies [20,21,28,29] (Table 3) using CANTAB AST, CNSVS ST & SAT, CNSVS SAT, and the Stroop test [45]. Two outcomes assessed in middle-aged or older individuals indicated statistically significant improvements (CANTAB AST congruency cost and CNSVS ST & SAT) [20,21], but no improvements were indicated on the other outcomes assessed for any age [28,29]. The correlation between the inhibition score of CNSVS ST & SAT and MP in older adults allocated to the intervention group was not statistically significant (*r* = 0.20, *p* = 0.10) [21], and correlations were not reported in the other studies [20,28,29]. A correlation with SL was not reported in all four studies [20,21,28,29]. SMDs of post-intervention in two studies were not statistically significant [20,28] and not reported in the other two studies [21,29].

#### 3.6.9. Shifting 

Five Shifting outcomes were measured in two studies [20,28] (Table 3) using Phonemic Fluency, Semantic Fluency, CNSVS SAT, and Verbal Fluency [46,47]. The outcome assessed for older individuals showed a statistically significant improvement within the group (Semantic Fluency) [28], but not correlated to SL (*r* = 0.03). The other outcomes assessed in middle-aged individuals were not statistically significant [20]. Correlations with MP were not reported in any studies. SMDs of the post-intervention in two studies were not statistically significant [20,28].

#### 3.6.10. Processing Speed

Ten Processing speed outcomes were measured in four studies [20,21,28,29] using CNSVS psychomotor speed, NES2 Pattern Comparison Test [21,29], CANTAB comprehension speed of response [20], and the Stroop Test (Mean RTs read word-black, Mean RTs read word-color, and Mean RTs name colors) [28]. No correlation was reported between processing speed and SL or MP. There was also no statistically significant improvement in all outcomes, and the SMD of the outcome was small.

### 3.7. Adverse Events

One study mentioned measuring adverse events by phone bimonthly but did not report the results [32]. Four studies did not report information on adverse events [20,21,28,29].

### 3.8. Astaxanthin Intervention

#### 3.8.1. Episodic Memory (visual stimuli)

Two outcomes were measured using the visual delayed recall test in CogHealth [30]. The response time was not changed among the groups. Only a high -dosage group showed significant improvement in the Delayed-Recall test score at 12 weeks of the intervention period compared to the baseline.

#### 3.8.2. Episodic Memory (Verbal Stimuli)

Two outcomes were measured in one study by using the Delayed Word Memory test with an original test battery [31]. The Delayed-Recall score of the Word Memory test improved compared with the initial within-group score in subjects aged >55 years [31].

#### 3.8.3. Short-term Memory/Working Memory

Six outcomes were measured in two studies by using the Visual Working Memory test with CogHealth [30], GMLT (Galton Maze Learning Test) [30], and Word Memory test using an original test battery [31]. Response time of Visual Working Memory test using CogHealth improved from baseline only in the 12 mg astaxanthin group [30]. GMLT performance at 8 weeks and 12 weeks of the treatment period in all groups was improved compared to the baseline performance [30]. Total errors in GMLT were reduced at 4, 8, 12 weeks of the intervention period in astaxanthin groups compared to the baseline [30]. The placebo group also showed a significant decrease in total errors in GMLT at 8 and 12 weeks compared to the baseline [30].

#### 3.8.4. Inhibition

Two outcomes were measured in one study [31] using the paper-pencil version Stroop test (Step 2, 4) [48]. For example, in the paper-pencil version Stroop test, “GREEN” word is presented in red color. In the step 2 (the reverse Stroop interference task), participants are asked to answer the ink color of the stimulus (correct answer is red). In the step 4 (the Stroop interference task), participants are asked to answer the color name of the stimulus (correct answer is green). The large sample study using the same paper-pencil version Stroop test reported the no gender differences of the reverse Stroop interference and the Stroop interference tasks in any age-range [49]. The result was consistent with the previous studies using oral response version and PC version Stroop tests [50,51].

The RCT showed that no significant differences were observed between or within groups [31].

#### 3.8.5. Attention

One outcome was measured in one study by divided attention using CogHealth [30]. No significant differences were observed between or within groups.

#### 3.8.6. Shifting

Three outcomes were measured in one trial by using the Semantic Fluency and Phonemic Fluency [31] (Table 2) tests. No significant differences were observed between or within groups.

#### 3.8.7. Processing Speed

Four outcomes were measured in two studies by (Table 2) [30,31] using the Stroop test Step 1, Stroop test Step 3, Simple reaction test and Choice reaction test. No significant differences were observed between or within groups.

### 3.9. Adverse Events

One study [30] reported that hematological tests, urinary tests, and oral consultation after 12 weeks of astaxanthin administration revealed no confirmed adverse effects. Another study [31] reported adverse events “unrelated” to astaxanthin intake, and physical measurements (i.e., blood and urine tests) showed minor changes.

### 3.10. Quality Assessment

#### 3.10.1. Methodological Quality in Included Studies

An assessment of the methodological quality of the included studies is presented in Table 4. The range of the quality assessment score was 9 to 11, with an average of 9.7 (SD = 0.76). All included studies had sufficient methodological quality. The scores of Item 9 (intention-to-treat analysis) and 14 (reporting CONSORT statement) were low. However, all studies fulfilled the methodological qualities of Item 1 (Random Allocation), Item 4 (Eligibly Criteria Specified), Item 5 (Blinded Outcome Assessor), Item 6 (Care Provider Blinded), and Item 7 (Patient Blinded). In addition, six studies fulfilled the requirements of Item 2 (Treatment Allocation Concealed) [20,21,28,29,30,32], Item 12 (Between-group Statistical Comparison) [20,21,29,30,31,32], and Item 13 (Reporting Dropouts) [21,28,29,30,31,32].

#### 3.10.2. Other Potential Sources of Bias

Power, 2018 was funded by the European Research Council. Lindberg 2018 was funded in part by Abbott Nutritional Products and the University of Georgia’s Bio-Imaging Research Center. Johnson 2008 was supported by USDA 1950-5100-065, Mead Johnson Nutritionals, and Martek Biosciences Corporation. Katagiri 2012 was supported by a grant from Yamaha Motors Co., Ltd. Two authors (M.H. and T.I.) of Hayashi 2018 were employees of JXTG Nippon Oil & Energy Corporation. Additionally, three studies were supported by Abbott Nutrition and used intervention supplements supplied by DSM Nutritional Products [21,29,32].

### 3.11. Excluded Studies

We excluded 13 relevant studies for using interventions other than carotenoids or its derivatives alone [52,53,54,55,56,57,58,59,60,61], not conducting cognitive assessment [62], and lack of a RCT [63].

## 4. Discussion

This study firstly summarized the beneficial effects of carotenoids on cognition in healthy adults. Five studies using lutein (and its isomers) and two studies using astaxanthin met the inclusion criteria of the SR. The SR revealed that three of the five studies with lutein reported significant benefit on several cognitive functions (visual episodic memory, verbal episodic memory, inhibition, and attention) compared to the placebo control group. However, the lutein consumption would not have beneficial effects on verbal episodic memory, working memory, reasoning, shifting, and processing speed. These results suggest that consumption of lutein would have a positive effect on cognitive functions in healthy adults. For astaxanthin, one of two studies showed significant improvements in memory performance compared to the placebo group. However, these findings should be interpreted with limitations due to the small number of studies. Therefore, we mainly discuss the result of lutein in the following sections.

For the benefits of lutein consumption, the SR obtained consistent results in visual episodic memory and inhibition between different age groups and between cognitive functional measurements. For visual episodic memory, two of the four studies that measured it showed significant performance improvement in young [29] and middle-aged adults [20]. For inhibition, two of the four studies that measured it showed significant performance improvements in middle-aged adults [20] and older adults [21]. Interestingly, the three studies which reported the cognitive benefits of lutein used the similar methods in terms of the intervention period (12 months) and the dose of lutein (10 mg per day). Based on these findings, 10 mg lutein consumption for twelve months can lead to improvement of visual episodic memory performance in young and middle-aged adults and improvement of inhibition performance in middle-aged and older adults.

On the contrary, inconsistent results were found in verbal episodic memory and attention domains. For verbal episodic memory, four of the five studies that measured verbal memory performance did not report significant group differences in young [29] and older adults [21,28,32], but one study showed significant group differences of in middle-aged adults [20]. Participants in the lutein group showed significantly fewer intrusion errors during the Delayed-Memory Recall compared to the control group [20]. However, it is important to note that the score of the intrusion errors at the baseline and the 12-month intervention period in both groups was almost zero (Table 3). In this case, the statistically significant finding would be clinically meaningful [20]. The study also measured several verbal episodic memory performances (e.g., immediate free recall and delayed free recall), but did not find any significant improvement in the memory performance. This study [20] used a similar dose of lutein (10 mg per day) and the same intervention period (12 months) to other three studies [21,29,32]. Based on these results, there seems to be no possibility that lutein consumption would lead to an improvement of verbal episodic memory performance.

Two of the five studies measured attention performance. Compared to the control group, a significant improvement was found in only older adults [21], but not in young adults [29]. The two studies used the same cognitive tests (CNSVS), dose of carotenoid (lutein: 10 mg per day and zeaxanthin: 2 mg per day), and intervention period (12 months). The difference in attention performance at the baseline would affect this inconsistent result. These studies calculated attention performance by combining the number of errors in attention tasks (a lower score means a few errors). The attention performance at the baseline was different between young adults (7.38) [29] and older adults (12.27) [21]. It could be difficult to find the improvement of attention performance in young adults because they had better attention at the baseline. Based on the inconsistent results, it is hard to conclude that lutein consumption had a benefit on attention performance. However, there is still a possibility that lutein consumption would improve attention performance because individuals with increased MPOD levels showed improved attention performance compared to non-increasers in young adults [29]. This suggests that attention performance would be improved in the young adults if the dose of lutein were increased. In addition, there is no study investigating the effect of lutein consumption on attention performance in middle-aged adults. In the future, it should be important to investigate the effect of lutein on cognitive function using a higher dose of lutein or middle-aged participants.

Several studies reported the significant correlation between cognitive functions (such as MP and SL) and carotenoid concentration. Previous cohort studies demonstrated the significant correlation between the MP and SL and a wide range of cognitive functions including visual episodic memory, inhibition, and attention in middle-aged and older adults [63,64,65]. The current results of this SR are consistent with these cohort studies. For example, the change of MP had a significant correlation with the improvement of memory performances, inhibition, attention in middle-aged and older adults [20,21]. One study reported that the change of MP and SL had a significant association with the decrease of memory errors in middle-aged adults [20]. These results directly indicated that the enhancement of carotenoid concentration by lutein consumption has a critical role in improvements of cognitive functions in middle-aged and older adults.

It is important to consider a biological mechanism of cognitive improvement by lutein consumption. Lutein and its isomers have roles of antioxidant and anti-inflammatory functions in the body [66]. A recent human study reported that lutein consumption for 6 months increased total antioxidant capacity (AOC) and brain-derived neurotrophic factor (BDNF) in young adults [67]. The level of BDNF is a biomarker of anti-inflammation because inflammation would affect the expression of BDNF [68]. In addition, the change of the BDNF and that of AOC were significantly correlated with cognitive functions, especially memory performance [67]. These results indicated that the antioxidant and anti-inflammatory functions of lutein and its isomers play a large role in improvements in cognitive functions.

Lutein and its isomers can cross the blood-brain barrier (BBB) [69]. Therefore, their anti-oxidant and anti-inflammatory functions would directly act in the human brain. Notably, lutein is selectively distributed in the frontal cortex, visual cortex, and hippocampus [70,71]. The concentration level of lutein and its isomers in the prefrontal cortex was higher than that in other regions [70]. The concentration of lutein was positively correlated with regional gray matter volume in the parahippocampal gyrus [72]. A cross-sectional brain imaging study using fMRI demonstrated that the concentration level of lutein was associated with brain activations in the inferior frontal gyrus (IFG) and the visual cortex [73]. A recent longitudinal fMRI study reported that one-year lutein consumption led to an increase of brain activation in the dorsolateral prefrontal cortex (DLPFC), anterior cingulate cortex (ACC), and hippocampus [32]. Previous neuroimaging studies reported that DLPFC, ACC, and inferior frontal gyrus are important to the inhibition process [74,75]. The medial temporal lobe, which included the hippocampus and parahippocampus, has an important role for memory functions [76,77]. These results suggest that lutein and its isomer would affect the memory and inhibition related brain regions.

For the benefits of astaxanthin on cognitive functions, only two studies were included in this SR [30,31]. The participants of the included studies were the middle-aged adults; however, different methodologies were used in the studies such as cognitive measurement, the intervention period (12 and 8 weeks), and the dose of astaxanthin (12 mg (6 mg) and 8 mg). One of the two studies using 8 mg astaxanthin reported significant improvement of verbal episodic memory performance after 8 weeks in middle-aged adults (less than 55 years old) [31]. Due to the small number of studies, it is difficult to conclude whether astaxanthin would have positive effects on cognitive functions. However, there is still a possibility that astaxanthin consumption has a positive effect on cognitive functions. Another study included in this SR reported that only a high dose astaxanthin group (12 mg) showed the significant pre-post improvement of working memory performance (reaction time) and delay visual episodic memory performance (accuracy) [30]. The low dose (6 mg) and placebo (0 mg) groups did not show any significant pre–post changes in working memory and delayed visual episodic memory performance [30]. This suggests that astaxanthin may be beneficial to memory function in middle-aged adults.

It is clear that the positive effects of these carotenoids on cognitive functions were determined on the basis of supplementation rather than normal daily consumptions. The previous studies demonstrated that the average adults consumed about 2 mg lutein per day [78,79]. It was lower than the dose of the supplementation (10 mg lutein). To take 10 mg lutein from vegetables and fruits, people need about 200 g every day [80]. For astaxanthin, if people take 4 mg astaxanthin in daily consumption, they need to eat about 600 to 2000 g salmon or seafood every day [81,82]. Two RCTs using astaxanthin were conducted in Japan. The National Health and Nutrition Survey in Japan in 2017 reported that average Japanese adults eat about 65 g seafood per day (https://www.mhlw.go.jp/bunya/kenkou/kenkou_eiyou_chousa.html). It was also lower than the dose of the supplementation (8 or 12 mg astaxanthin). These results indicate that it is not easy to take enough lutein and astaxanthin to affect cognitive functions from daily consumption. The supplementation of lutein or astaxanthin led to improvements of cognitive functions in the RCT.

The limitations of this SR are the inclusion of only seven RCT studies and the use of a wide range of cognitive functional measures among included studies (Table 2). It would be difficult to summarize the cognitive domain among studies. Due to the small number of studies and different cognitive functional measurements, it is hard to make a clear conclusion about the benefits of carotenoid intakes on cognitive functions. More RCT studies with lutein and astaxanthin using similar and common cognitive functional tests are needed to make a clear conclusion.

Seven RCT studies in the SR did not report adverse events related to lutein and astaxanthin. A previous meta-analysis reported that overdose of antioxidant increases the risk of a health problem [83]. It must be noted that supplementation with antioxidants should be required delicate handling because of the risk of overdosing poses.

## 5. Conclusions

We firstly conducted the SR for RCTs to investigate the benefits of carotenoids intakes on cognitive functions. From seven RCT studies (five studies using lutein and two studies using astaxanthin), three studies using lutein reported significant improvements of several cognitive functions (visual episodic memory, verbal episodic memory, inhibition, attention). The SR revealed that lutein selectively leads to improvement of visual episodic memory in young and middle-aged adults and inhibition in the middle-aged and older adults. For astaxanthin, one study reported significant improvement of verbal episodic memory performance in middle-aged adults. The SR indicates that carotenoid (lutein and astaxanthin) would have positive impacts on cognitive functions among different age groups.

## Figures and Tables

**Table 1:** Characteristics of included studies.

Lead Author; Year; Country	Study Design, Duration	Sample size (Female)	Age (mean ± SD)	Health Status	Cognitive Status	Intervention (Timing or Method)	Control (Contents)
**Power; 2018; Ireland**	A parallel-group, double-blind, placebo-controlled, block-randomized clinical trial, 12 months	91 (48%) P: 46 A: 45	P: 46.43 ± 13.21 A: 44.38 ± 11.57	Low MP volume without the retinal disease, no consumption of L and/or Z and/or MZ supplement	No impairment	L: 10 mg/d MZ: 10 mg/d Z: 2 mg/d (with a meal)	Placebo (capsule containing sunflower oil)
**Lindberg; 2017; USA**	A single-site, double-blind RCT, 12 months	44 (59%) P: 14 A: 30	P: 70.43 ± 5.43 A: 72.43 ± 6.48	Community-dwelling older adults, good overall health, no consumption of xanthophyll supplement	*n*/R	L: 10 mg/d Z: 2 mg/d (*n*/R)	Placebo (*n*/R)
**Renzi-Hammond; 2017; USA**	A randomized, double-masked, placebo-controlled trial, 12 months	51 (43%) P: 14 A: 37	P: 20.5 ± 1.91 A: 21.5 ± 2.69	Healthy young college students, no consumption of the supplement	No impairment	L: 10 mg/d Z: 2 mg/d (with the highest fat meal)	Placebo (*n*/R)
**Hammond; 2017; USA**	The double-masked, randomized, placebo-controlled trial, 12 months	51 (59%) P: 15 A: 36	P: 70.93 ± 5.70 A: 72.51 ± 6.24	Healthy community-dwelling older adults, no consumption of L&Z supplement	No impairment	L: 10 mg/d Z: 2 mg/d (with the highest fat meal)	Placebo (*n*/R)
**Johnson; 2008; USA**	Randomized, double-blind, intervention trial, 4 months	49 (100%) P: 10 D: 14 L: 11 D+L: 14	P: 68.0 ± 1.2 D: 68.5 ± 1.3 L: 66.7 ± 1.9 D+L: 68.6 ± 1.3	Healthy, non-smoking older women, no consumption of carotenoids supplement	No impairment	L: 12 mg/d (with nutritional energy drink)	Placebo (*n*/R)
**Katagiri; 2012; Japan**	The randomized double-blind placebo-controlled study, 12 weeks	89 (*n*/R) HAx: 29 LAx: 29 P: 31	HAx: 51.5 ± 5.7 LAx: 51.1 ± 5.9 P: 51.6 ± 5.3	Healthy men and women	Complaints of age-related forgetfulness	HAx: A 12 mg/d LAx: A 6 mg/d (after breakfast or lunch or supper)	Placebo (a pill containing corn oil)
**Hayashi; 2018; Japan**	Randomized, double-blind, placebo-controlled, parallel inter-group comparison 8 weeks	54 (54%) P: 26 Ax: 28	P: 54.4 ± 6.0 Ax: 56.0 ± 5.2	Healthy men and women	No impairment	Ax 8 mg/d (after breakfast and dinner)	Placebo (jelly without Ax)

Note: RCT: Randomized control trial. P: Placebo control group. A: Active intervention group. D: DHA supplementation group. L: Lutein supplementation group. D+L: DHA + Lutein supplementation group. HAx: High astaxanthin supplementation group. LAx: Low astaxanthin supplementation group. MP: Macular pigment. L: Lutein. Z: Zeaxanthin. MZ: Meso-zeaxanthin. Ax: Astaxanthin. *n*/R: Not reported.

**Table 2:** Description of measurements, analysis, and intervention effects of cognitive function.

Lead author; Year; Country	Test Battery	Tasks for Calculating the Index Score	Domain of Cognitive Functions	Measurement Indices	Statistical Analysis Methods	Results	
						Analytical Objects	Intervention Effects
Power; 2018; Ireland	CANTAB	MOT	Processing speed	Mean latency	rANOVA (Only time effects and time-group interaction effects are shown)	Value	*n*/R
				Mean total correct		Value	*n*/R
				Mean total errors		Value	*n*/R
		AST	Inhibition	AST correct latency		Value	0
				AST percent correct		Value	0
				AST congruency cost		Value	+
		VRM	Episodic memory (verbal stimuli)	VRM Trial 1 immediate free recall		Value	*n*/R
				VRM Trial 2 immediate free recall		Value	*n*/R
				VRM Trial 3 immediate free recall		Value	*n*/R
				VRM Total immediate free recall		Value	*n*/R
				VRM Trial 1 intrusion errors		Value	*n*/R
				VRM Trial 2 intrusion errors		Value	*n*/R
				VRM Trial 3 intrusion errors		Value	*n*/R
				VRM Learning slope		Value	*n*/R
				VRM delayed free recall		Value	*n*/R
				VRM delayed intrusion errors		Value	+
				VRM delayed recognition total		Value	*n*/R
				VRM recognition false positives		Value	*n*/R
		PAL	Episodic memory (visual stimuli)	PAL memory score		Value	+
				PAL total errors		Value	*n*/R
				PAL total errors stage 6		Value	+
	“FAS” and “Animal” test	FAS test	Shifting	Sum of the number of words beginning with the letters F, A, and S generated with a 1-min time limit per letter		Value	0
		Animal test	Shifting	The number of animal names generated with a 1-min time limit		Value	*n*/R
Lindberg; 2018; USA	WTAR	WTAR	Global cognition	Number of words correctly pronounced	2 × 2 mixed-design ANOVA	Value	*n*/R
	fMRI task	Wechsler memory scale paired associates learning test	Episodic memory (verbal stimuli)	Number of cued recall	*t*-test	Value	0
Renzi-Hammond; 2017; USA	CNSVS	VBM	Episodic memory (verbal stimuli)	Verbal memory score (VBM correct hits (immediate) + VBM correct – passes (immediate) + VBM correct hits (delay) + VBM correct − passes (delay))	RCI	Delta	*n*/R
		VIM	Episodic memory (visual stimuli)	Visual memory score (VIM correct hits (immediate) + VIM correct − passes (immediate) + VIM correct hits (delay) + VIM correct − passes (delay))		Delta	+
		NVRT	Reasoning	Reasoning score (NVRT correct responses − NVRT errors)		Delta	+*(“MPOD increaser”)
		SAT	Shifting	Executive function score (SAT correct responses − SAT errors)		Delta	*n*/R
		FTT, SDC	Processing speed	Psychomotor speed score (FTT right response + FTT left response + SDC corrects)		Delta	*n*/R
		ST, SAT, CPT	Attention	Complex Attention score (ST errors + SAT errors + CPT errors + CPT correct − passes)		Delta	+*(“MPOD increaser”)
		ST, SAT	Inhibition	Cognitive Flexibility score (SAT correct responses − SAT error − ST error)		Delta	*n*/R
Hammond; 2017; USA	CNSVS	VBM	Episodic memory (verbal stimuli)	Verbal memory score (VBM correct hits (immediate) + VBM correct − passes (immediate) + VBM correct hits (delay) + VBM correct − passes (delay))	RCI	Delta	*n*/R
		VIM	Episodic memory (visual stimuli)	Visual memory score (VIM correct hits (immediate) + VIM correct − passes (immediate) + VIM correct hits (delay) + VIM correct − passes (delay))		Delta	*n*/R
		NVRT	Reasoning	Reasoning score (NVRT correct responses − NVRT errors)		Delta	*n*/R
		SAT	Shifting	Executive function score (SAT correct responses − SAT errors)		Delta	+‡
		FTT, SDC	Processing speed	Psychomotor speed score (FTT right response + FTT left response + SDC corrects)		Delta	*n*/R
		ST, SAT, CPT	Attention	Complex Attention score (ST errors + SAT errors + CPT errors + CPT correct-passes)		Delta	+
		ST, SAT	Inhibition	Cognitive Flexibility score (SAT correct responses − SAT error − ST error)		Delta	+
		VBM, VIM, FTT, SDC, ST, SAT, CPT	Global cognition	Neurocognitive Index (NCI) (Average of following five scores; Composite Memory, Psychomotor Speed, Reaction Time, Complex Attention, and Cognitive Flexibility)		Delta	*n*/R
Johnson; 2008; USA	Original test battery	Verbal Fluency	Shifting	Number of words from a category during a 1-min	Student’s paired *t*-test	Value	+†(L)
		Digit Span Forward and Backward	Short-term memory/working memory	Forward digit span length		Value	0
				Forward digit span total		Value	0
				Backward digit span length		Value	0
				Backward digit span total		Value	0
		Shopping List Task	Episodic memory (verbal stimuli)	Trial 1 items recalled (max. 10)		Value	0
				Trials to learn list (max. 4)		Value	0
				Delayed recall (max. 10)		Value	0
		Word List Memory Test (computer version)	Episodic memory (verbal stimuli)	Trial 1 items recalled (max. 10)		Value	0
				Trials to learn list (max. 6)		Value	0
				Delayed recall (max. 10)		Value	0
		Memory in Reality (MIR) Apartment Test	Episodic memory (visual stimuli)	Delayed recall (max. 10)		Value	0
				Location recall (max. 10)		Value	0
		NES2 Pattern Comparison Test	Processing speed	Number correct (max.15)		Value	0
				Mean response time-correct (s)		Value	-
		Stroop Test	Processing speed	Mean RT, read words-black (ms)		Value	0
			Processing speed	Mean RT, read words-color (ms)		Value	0
			Processing speed	Mean RT, name colors (ms)		Value	0
			Inhibition	Mean RT, name color-words (ms)		Value	0
			Inhibition	Total RT, interference (NC-C) (s)		Value	0
Katagiri; 2012; Japan	CogHealth	Simple reaction test	Processing speed	Response time (ms)	Two-way factorial ANOVA, adjusted for age and sex (between-group) One-way repeated measure ANOVA, adjusted for age and sex (inter-group) Bonferroni correction (multiple comparisons)	Value	0
		Choice reaction test		Response time (ms)		Value	0
		Working memory test	Short-term memory/working memory	Response time (ms)		Value	+†(HAx)
				Accuracy (%)		Value	0
		Delayed recall test	Episodic memory (visual stimuli)	Response time (ms)		Value	0
				Accuracy (%)		Value	+†(HAx)
		Divided attention test	Attention	Response time (ms)		Value	0
	GMLT	GMLT test	Short-term memory/working memory	Total duration (s)		Value	+†(HAx, LAx, P)
				Total errors		Value	+†(HAx, LAx, P)
Hayashi; 2018; Japan	Original test battery	Word memory test	Short-term memory/working memory	Immediate recall	Two-way repeated measures ANOVA	Delta	0
				Immediate recall + cued recall		Delta	0
			Episodic memory (verbal stimuli)	Recall after 5 min		Delta	+*(age < 55)
				Recall after 5 min + cued recall		Delta	0
		Verbal fluency test	Shifting	Names of vegetables		Delta	0
				Words that begin with “a”		Delta	0
				Animal words		Delta	0
		Stroop test	Processing speed	Stroop test Step 1		Delta	0
			Inhibition	Stroop test Step 2		Delta	0
			Processing speed	Stroop test Step 3		Delta	0
			Inhibition	Stroop test Step 4		Delta	0

Note: CANTAB: Cambridge Neuropsychological Test Automated Battery. WTAR: Wechsler Test of Adult Reading. CNSVS: CNS Vital Signs testing platform. GMLT: Groton Maze Leaning Test. MOT: Motor screening task. AST: Attention switching task. VRM: Verbal recognition memory. PAL: Paired associated learning. VBM: Verbal memory test. VIM: Visual memory test. NVRT: Non-verbal reasoning test. SAT: Shifting attention test. FTT: Finger tapping test. SDC: Symbol-digit coding test. ST: Stroop test CNSVS ver. CPT: Continuous performance task. rANOVA: Repeated measures analyses of variance. RCI: Reliable Change Index. ANOVA: analyses of variance. Value: Measured values were used to detect intervention effects. Delta: Change values between baseline and various measurement points were used to detect intervention effects. HA: High Astaxanthin group. LA: Low Astaxanthin group. P: Placebo group. *n*/R: Not Reported. +: there is a significant effect in comparison between groups. +*: there is a significant effect in subgroup analysis. +†: there is a significant effect in comparison between baseline and after intervention measure. +‡: there is a trend in between groups. 0: No change between placebo group and intervention group. −: the control group improved cognitive functions compared to the target intervention group.

**Table 3:** Correlation between serum lutein (SL) or macular pigment (MP) and cognitive function.

Cognitive Function	Lead Author; Year; Country	Measurement Indices (Task Name)	Correlation with Task (*p*-Value)		Task Score, Mean ± SD (Pre) [Post]{Change}		Observed Effect	SMD [95%CI] (Bigger Number Shows Active Favors)		
			SL	MP	Active	Placebo	Statistical Method (*p*-Value)	P pre vs. A pre (*p*-Value)	P Post vs. A Post (*p*-Value)	P Change vs. A Change (*p*-Value)
Episodic memory (visual stimuli)	Power; 2018; Ireland	PAL memory score (PAL, CANTAB)	*r* = 0.159 (*p* = 0.226)	*r* = 0.219 (*p* = 0.078)	(18.91 ± 4.96) [20.77 ± 4.57] {1.86 ± 4.72}	(21.26 ± 3.52) [20.32 ± 4.57] {−0.94 ± 3.48}	rANOVA (time effect *p* = 0.376, time × group effect *p* = 0.009)	0.55 [0.05, 1.04] (*p* = 0.03)	0.10 [−0.39, 0.58] (*p* = 0.69)	0.66 [1.16, 0.16] (*p* = 0.009)
		PAL total errors (PAL, CANTAB)	*n*/R	*n*/R	(21.50 ± 28.20) [*n*/R] {*n*/R}	(17.22 ± 16.93) [*n*/R] {*n*/R}	rANOVA (*n*.S.)	−0.18 [−0.59, 0.23] (*p* = 0.38)	-	-
		PAL errors adjusted for stage 6 (PAL, CANTAB)	*r* = −0.346 (*p* = 0.006)	*r* = −0.342 (*p* = 0.005)	(6.78 ± 7.10) [3.17 ± 4.52] {−3.61 ± 7.01}	(4.19 ± 3.82) [4.48 ± 4.89] {0.29 ± 5.79}	rANOVA (time effect *p* = 0.040, time × group effect *p* = 0.017)	−0.44 [−0.93, 0.05] (*p* = 0.08)	0.28 [−0.21, 0.76] (*p* = 0.28)	0.60 [0.10, 1.09] (*p* = 0.02)
	Renzi-Hammond; 2017; USA	Visual memory (VIM, CNSVS)	*n*/R	*n*/R	(*n*/R) [*n*/R] {9.43 ± *n*/R}	(*n*/R) [*n*/R] {4.93 ± *n*/R}	RCI (RCI active = 6.77 vs. RCI placebo = 1.88, *p* < 0.04)	-	-	-
	Hammond; 2017; USA	Visual memory (VIM, CNSVS)	*n*/R	*r* = 0.24 (*p* = 0.09, in A only)	(41.03 ± 6.68) [*n*/R] {*n*/R}	(43.87 ± 5.18) [*n*/R] {*n*/R}	RCI (*n*.S.)	-	-	-
	Johnson; 2008; USA	Delayed recall (MIR apartment test)	−0.16 (*n*.S.)	*n*/R	(8.3 ± 1.6) [8.6 ± 2.1] {*n*/R}	(9.3 ± 0.8) [9.4 ± 0.7] {*n*/R}	Student’s paired *t*-test (*n*.S.)	0.75 [−0.15, 1.64] (*p* = 0.10)	0.48 [−0.39, 1.35] (*p* = 0.28)	-
		Location recall (MIR apartment test)	*n*/R	*n*/R	(9.5 ± 1.0) [9.5 ± 0.8] {*n*/R}	(9.7 ± 0.7) [9.7 ± 0.7] {*n*/R}		0.22 [−0.64, 1.08] *(p* = 0.62)	0.25 [−0.61, 1.12] (*p* = 0.56)	-
Episodic memory (verbal stimuli)	Power; 2018; Ireland	VRM Trial 1 immediate free recall (VRM, CANTAB)	*n*/R	*n*/R	(8.17 ± 1.75) [8.69 ± 1.67] {0.52 ± 1.68}	(8.57 ± 1.59) [8.73 ± 1.84] {0.17 ± 1.58}	rANOVA (*n*.S.)	−0.24 [−0.72, 0.25] (*p* = 0.34)	−0.02 [−0.51, 0.46] (*p* = 0.93)	0.21 [−0.27, 0.70] (*p* = 0.39)
		VRM Trial 2 immediate free recall (VRM, CANTAB)	*n*/R	*n*/R	(9.83 ± 1.48) [10.11 ± 2.30] {0.28 ± 1.67}	(10.10 ± 1.24) [10.60 ± 1.57] {0.50 ± 1.80}		−0.19 [−0.68, 0.29] (*p* = 0.43)	−0.24 [−0.73, 0.24] (*p* = 0.33)	−0.13 [−0.61, 0.36] (*p* = 0.61)
		VRM Trial 3 immediate free recall (VRM, CANTAB)	*n*/R	*n*/R	(10.71 ± 1.72) [10.51 ± 2.13] {−0.20 ± 1.35}	(10.80 ± 1.10) [11.03 ± 1.13] {0.23 ± 1.17}		−0.06 [−0.55, 0.43] (*p* = 0.81)	−0.29 [−0.79, 0.20] (*p* = 0.24)	−0.33 [−0.83, 0.16] (*p* = 0.18)
		VRM Total immediate free recall (VRM, CANTAB)	*n*/R	*n*/R	(28.67 ± 4.51) [29.03 ± 5.52] {0.36 ± 3.22}	(29.47 ± 3.17) [30.37 ± 3.77] {0.90 ± 3.25}		−0.2 [−0.69, 0.29] (*p* = 0.42)	−0.28 [−0.76, 0.21] (*p* = 0.27)	−0.17 [−0.65, 0.32] (*p* = 0.51)
		VRM Trial 1 intrusion errors (VRM, CANTAB)	*n*/R	*n*/R	(0.11 ± 0.32) [0.11 ± 0.32] {0 ± 0.48}	(0.17 ± 0.46) [0.20 ± 0.55] {0.03 ± 0.49}		−0.15 [−0.64, 0.33] (*p* = 0.54)	−0.20 [−0.69, 0.28] (*p* = 0.54)	−0.06 [−0.55, 0.42] (*p* = 0.80)
		VRM Trial 2 intrusion errors (VRM, CANTAB)	*n*/R	*n*/R	(0.08 ± 0.28) [0.06 ± 0.23] {−0.02 ± 0.38}	(0.03 ± 0.18) [0.20 ± 0.48] {0.17 ± 0.46}		0.21 [−0.28, 0.69] (*p* = 0.41)	−0.38 [−0.87, 0.11] (*p* = 0.13)	−0.45 [−0.94, 0.04] (*p* = 0.07)
		VRM Trial 3 intrusion errors (VRM, CANTAB)	*n*/R	*n*/R	(0.06 ± 0.23) [0 ± 0] {−0.06 ± 0.23}	(0.07 ± 0.36) [0.13 ± 0.50] {0.06 ± 0.63}		−0.03 [−0.52, 0.45] (*p* = 0.89)	-	−0.26 [−0.75, 0.23] (*p* = 0.30)
		VRM Learning slope (VRM, CANTAB)	*n*/R	*n*/R	(2.54 ± 2.09) [1.89 ± 1.84] {−0.65 ± 1.89}	(2.20 ± 1.32) [2.30 ± 1.64] {0.10 ± 1.63}		0.20 [−0.29, 0.68] (*p* = 0.43)	−0.23 [−0.72, 0.26] (*p* = 0.35)	−0.42 [−0.92, 0.07] (*p* = 0.09)
		VRM delayed free recall (VRM, CANTAB)	*n*/R	*n*/R	(9.07 ± 3.25) [9.92 ± 1.72] {0.85 ± 3.22}	(10.04 ± 1.43) [10.08 ± 2.73] {0.04 ± 2.18}		−0.38 [−0.94, 0.19] (*p* = 0.19)	−0.07 [−0.62, 0.49] (*p* = 0.81)	0.29 [−0.27, 0.85] (*p* = 0.31)
		VRM delayed intrusion errors (VRM, CANTAB)	*r* = −0.189 (*p* = 0.220)	*r* = −0.306 (*p* = 0.033)	(0.12 ± 0.33) [0.04 ± 0.20] {−0.08 ± 0.39}	(0.00 ± 0.00) [0.21 ± 0.51] {0.21 ± 0.51}	rANOVA (time effect *p* = 0.309, time × group effect *p* = 0.030)	−0.5 [−1.06, 0.07] (*p* = 0.08)	0.44 [−0.12, 1.00] (*p* = 0.13)	0.63 [0.06, 1.20] (*p* = 0.03)
		VRM delayed recognition total (VRM, CANTAB)	*n*/R	*n*/R	(23.85 ± 0.36) [23.82 ± 0.52] {−0.03 ± 0.52}	(23.83 ± 0.38) [23.87 ± 0.43] {0.04 ± 0.49}	rANOVA (*n*.S.)	0.05 [−0.44, 0.54] (p = 0.83)	−0.1 [−0.59, 0.39] (p = 0.68)	−0.14 [−0.63, 0.35] (p = 0.59)
		VRM recognition false positives (VRM, CANTAB)	*n*/R	*n*/R	(0.03 ± 0.17) [0.09 ± 0.38] {0.06 ± 0.34}	(0.10 ± 0.31) [0.07 ± 0.37] {−0.03 ± 0.32}		0.27 [−0.22, 0.76] (*p* = 0.29)	0.05 [−0.44, 0.54] (*p* = 0.83)	−0.28 [−0.78, 0.21] (*p* = 0.26)
	Lindberg; 2018; USA	Wechsler memory scale paired associates learning test	*n*/R	*n*/R	(8.87 ± 1.50) [8.80 ± 2.16] {*n*/R}	(9.36 ± 0.75) [8.21 ± 2.29] {*n*/R}	t-test, 2 × 2 mixed design ANOVA (*n*.S.)	−0.37 [−1.01, 0.27] (*p* = 0.26)	0.26 [−0.37,0.90] (*p* = 0.42)	-
	Renzi-Hammond; 2017; USA	Verbal memory (VEM, CNSVS)	*n*/R	*n*/R	(*n*/R) [*n*/R] {*n*/R}	(*n*/R) [*n*/R] {*n*/R}	RCI (*n*.S.)	-	-	-
	Hammond; 2017; USA	Verbal memory (VEM, CNSVS)	*n*/R	*r* = 0.31 (*p* = 0.07, “MPOD increaser”)	(49.91 ± 5.66) [*n*/R] {*n*/R}	(52.67 ± 5.29) [*n*/R] {*n*/R}	RCI (*n*.S.)	−0.49 [−1.10, 0.12] (*p* = 0.12)	-	-
	Johnson; 2008; USA	Trial 1 items recalled (max. 10) (Shopping List Task)	*n*/R	*n*/R	(6.9 ± 1.8) [6.5 ± 2.1] {*n*/R}	(6.5 ± 1.2) [7.7 ± 1.5] {*n*/R}	Student’s paired t-test (*n*.S.)	0.25 [−0.61, 1.11] (*p* = 0.57)	−0.63 [−1.51, 0.26] (*p* = 0.16)	-
		Trials to learn list (max. 6) (Shopping List Task)	0.30 (*p* < 0.05)	*n*/R	(4.2 ± 1.5) [3.9 ± 1.4] {*n*/R}	(3.0 ± 0.8) [2.8 ± 0.9] {*n*/R}		−0.94 [−1.86, −0.03] (*p* = 0.04)	−0.89 [−1.79, 0.02] (*p* = 0.06)	-
		Delayed recall (max. 10) (Shopping List Task)	*n*/R	*n*/R	(8.3 ± 1.9) [7.6 ± 3.0] {*n*/R}	(9.5 ± 0.9) [9.5 ± 0.7] {*n*/R}		0.25 [−0.61, 1.11] (*p* = 0.09)	−0.82 [−1.72, 0.08] (*p* = 0.07)	-
		Trial 1 items recalled (max. 10) (Word List Memory Test)	*n*/R	*n*/R	(5.8 ± 1.8) [5.8 ± 1.8] {*n*/R}	(6.2 ± 1.3) [6.6 ± 1.8] {*n*/R}		−0.24 [−1.10, 0.62] (*p* = 0.58)	−0.43 [−1.29, 0.44] (*p* = 0.34)	-
		Trials to learn list (max. 4) (Word List Memory Test)	−0.04 (*n*.S.)	*n*/R	(3.4 ± 0.7) [3.5 ± 0.8] {*n*/R}	(3.1 ± 0.9) [2.8 ± 0.9] {*n*/R}		−0.36 [−1.22, 0.51] (*p* = 0.42)	−0.79 [−1.69, 0.10] (*p* = 0.08)	-
		Delayed recall (max. 10) (Word List Memory Test)	*n*/R	*n*/R	(6.8 ± 2.9) [7.6 ± 2.4] {*n*/R}	(8.1 ± 1.1) [8.3 ± 1.8] {*n*/R}		−0.56 [−1.43, 0.32] (*p* = 0.21)	−0.31 [−1.18, 0.55] (*p* = 0.48)	-
Short-term memory/working memory	Johnson; 2008; USA	Forward digit span length	*n*/R	*n*/R	(6.6 ± 1.2) [7.0 ± 1.5] {*n*/R}	(7.2 ± 1.2) [7.2 ± 1.4] {*n*/R}	Student’s paired *t*-test (*n*.S.)	−0.48 [−1.35, 0.39] (*p* = 0.28)	−0.13 [−0.99, 0.73] (*p* = 0.76)	-
		Forward digit span total	*n*/R	*n*/R	(8.1 ± 2.3) [8.7 ± 2.5] {*n*/R}	(9.7 ± 2.5) [9.0 ± 2.4] {*n*/R}		−0.64 [−1.52, 0.24] (*p* = 0.28)	−0.12 [−0.97, 0.74] (*p* = 0.79)	-
		Backward digit span length	*n*/R	*n*/R	(5.1 ± 1.6) [4.7 ± 1.4] {*n*/R}	(5.9 ± 1.4) [5.8 ± 1.7] {*n*/R}		−0.51 [−1.38, 0.36] (*p* = 0.25)	−0.68 [−1.57, 0.20] (*p* = 0.13)	-
		Backward digit span total	*n*/R	*n*/R	(7.5 ± 3.1) [6.9 ± 2.7] {*n*/R}	(8.2 ± 2.7) [8.4 ± 3.3] {*n*/R}		−0.23 [−1.09, 0.63] (*p* = 0.60)	−0.48 [−1.35, 0.39] (*p* = 0.28)	-
Reasoning	Renzi-Hammond; 2017; USA	Reasoning (NVRT, CNSVS)	*n*/R	*n*/R	(*n*/R) [*n*/R] {*n*/R}	(*n*/R) [*n*/R] {*n*/R}	RCI (RCI “MPOD increase” = 1.94 vs. RCI “no change” = 0.18, *p* < 0.05*)	-	-	-
	Hammond; 2017; USA	Reasoning (NVRT, CNSVS)	*n*/R	*r* = 0.45 (*p* = 0.04)	(2.97 ± 3.95) [*n*/R] {*n*/R}	(2.47 ± 3.83) [*n*/R] {*n*/R}	RCI (*n*.S.)	0.13 [−0.48, 0.73] (*p* = 0.68)	-	-
Attention	Renzi-Hammond; 2017; USA	Complex attention (ST & SAT & CPT, CNSVS)	*n*/R	*n*/R	(*n*/R) [*n*/R] {*n*/R}	(*n*/R) [*n*/R] {*n*/R}	RCI (RCI “MPOD increase” = 2.02 vs. RCI “no change” = 0.00, *p* < 0.04)	-	-	-
	Hammond; 2017; USA	Complex attention (ST & SAT & CPT, CNSVS)	*n*/R	*r* = −0.31 (*p* = 0.04, in A only)	(13.12 ± 11.12) [*n*/R] {*n*/R}	(10.21 ± 7.21) [*n*/R] {*n*/R}	RCI (RCI active = 3.71 vs. RCI placebo = 0.34, *p* < 0.02)	0.28 [−0.32, 0.89] (*p* = 0.36)	-	-
Inhibition	Power; 2018; Ireland	AST correct latency (AST, CANTAB)	*n*/R	*n*/R	(832 ± 191.86) [751.63 ± 191.70] {−80.37 ± 132.98}	(841.41 ± 158.95) [775.38 ± 217.58] {−66.03 ± 167.79}	rANOVA (time effect *p* < 0.001, time × group effect *p* = 0.695)	0.05 [−0.42,0.53] (*p* = 0.83)	0.12 [−0.36,0.59] (*p* = 0.83)	−0.09 [−0.57, 0.38] (*p* = 0.70)
		AST percent correct (AST, CANTAB)	*n*/R	*n*/R	(93.71 ± 8.14) [96.84 ± 3.49] {3.13 ± 6.94}	(93.89 ± 7.57) [95.02 ± 7.61] {1.13 ± 4.10}	rANOVA (time effect *p* = 0.004, time × group effect *p* = 0.164)	−0.02 [−0.50, 0.45] (*p* = 0.93)	0.31 [−0.17, 0.79] (*p* = 0.20)	0.34 [−0.14, 0.82] (*p* = 0.17)
		AST congruency cost (AST, CANTAB)	*n*/R	*n*/R	(98.46 ± 103.1) [94.43 ± 70.01] {−4.03 ± 93.04}	(128.1 ± 99.07) [72.74 ± 86.65] {−55.36 ± 110.56}	rANOVA (time effect *p* = 0.019, time × group effect *p* = 0.041)	−0.29 [−0.77, 0.19] (*p* = 0.24)	0.27 [−0.20, 0.75] (*p* = 0.26)	0.5 [0.02, 0.99] (*p* = 0.104)
	Renzi-Hammond; 2017; USA	Cognitive flexibility (ST & SAT, CNSVS)	*n*/R	*n*/R	(*n*/R) [*n*/R] {*n*/R}	(*n*/R) [*n*/R] {*n*/R}	RCI (*n*.S.)	-	-	-
	Hammond; 2017; USA	Cognitive flexibility (ST & SAT, CNSVS)	*n*/R	*r* = 0.20 (*p* = 0.10)	(30.32 ± 19.09) [*n*/R] {*n*/R}	(35.5 ± 16.01) [*n*/R] {*n*/R}	RCI (RCI active = 6.31 vs. RCI placebo = 0.84, *p* < 0.04)	−0.28 [−0.88, 0.33] (*p* = 0.37)	-	-
	Johnson; 2008; USA	Mean RT, name colors – words (Stroop test)	*n*/R	*n*/R	(1492 ± 329) [1462 ± 221] {*n*/R}	(1419 ± 308) [1413 ± 508] {*n*/R}	Student’s paired *t*-test (*n*.S.)	−0.22 [−1.08, 0.64] (*p* = 0.62)	−0.12 [−0.98, 0.74] (*p* = 0.78)	-
		Total RT, interference (NC-C) (Stroop test)	*n*/R	*n*/R	(24.2 ± 10.9) [22.4 ± 7.1] {*n*/R}	(25.0 ± 14.8) [23.1 ± 22.0] {*n*/R}		0.06 [−0.80, 0.92] (*p* = 0.89)	0.04 [−0.81, 0.90] (*p* = 0.92)	-
Shifting	Power; 2018; Ireland	Phonemic (letter) fluency (FAS test)	*n*/R	*n*/R	(44.44 ± 15.49) [50.11 ± 15.55] {5.67 ± 7.85}	(40.23 ± 11.84) [45.93 ± 10.91] {5.70 ± 7.88}	rANOVA (time effect *p* < 0.001, time × group effect *p* = 0.986)	0.30 [−0.19, 0.79] (*p* = 0.23)	0.30 [−0.18, 0.79] (*p* = 0.22)	0.00 [−0.49, 0.48] (*p* = 0.99)
		Semantic (category) fluency (Animal test)	*n*/R	*n*/R	(23.72 ± 6.96) [24.25 ± 6.97] {0.53 ± 5.20}	(19.87 ± 3.86) [21.30 ± 3.66] {1.43 ± 3.57}	rANOVA (*n*.S.)	0.7 [0.28, 1.13] (*p* = 0.001)	0.51 [0.02, 1.00] (*p* = 0.04)	−0.2 [−0.68, 0.29] (*p* = 0.43)
	Renzi-Hammond; 2017; USA	Executive function (SAT, CNSVS)	*n*/R	*n*/R	(*n*/R) [*n*/R] {*n*/R}	(*n*/R) [*n*/R] {*n*/R}	RCI (*n*.S.)	-	-	-
	Hammond; 2017; USA	Executive function (SAT, CNSVS)	*n*/R	*n*/R	(32.33 ± 18.33) [*n*/R] {*n*/R}	(35.87 ± 15.90) [*n*/R] {*n*/R}	RCI (RCI active = 5.64 vs. RCI placebo = 1.27, *p* = 0.07)	−0.2 [−0.80, 0.41] (*p* = 0.52)	-	-
	Johnson; 2008; USA	Verbal Fluency (Verbal Fluency)	0.03 (*n*.S.)	*n*/R	(11.3 ± 5.1) [15.5 ± 5.5] {*n*/R}	(12.9 ± 6.2) [13.8 ± 3.5] {*n*/R}	Student’s paired *t*-test (*p* < 0.05)	−0.27 [−1.13, 0.59] (*p* = 0.54)	0.35 [−0.51,1.21] (*p* = 0.43)	-
Processing speed	Power; 2018; Ireland	Mean latency (CANTAB, MOT)	*n*/R	*n*/R	(*n*/R) [*n*/R] {*n*/R}	(*n*/R) [*n*/R] {*n*/R}	rANOVA (*n*.S.)	-	-	-
		Mean total correct (CANTAB, MOT)	*n*/R	*n*/R	(*n*/R) [*n*/R] {*n*/R}	(*n*/R) [*n*/R] {*n*/R}		-	-	-
		Mean total errors (CANTAB, MOT)	*n*/R	*n*/R	(*n*/R) [*n*/R] {*n*/R}	(*n*/R) [*n*/R] {*n*/R}		-	-	-
	Renzi-Hammond; 2017; USA	Psychomotor speed score (CNSVS, FTT+SDC)	*n*/R	*n*/R	(*n*/R) [*n*/R] {*n*/R}	(*n*/R) [*n*/R] {*n*/R}	RCI (*n*.S.)	-	-	-
	Hammond; 2017; USA	Psychomotor speed score (CNSVS, FTT+SDC)	*n*/R	*n*/R	(145.5 ± 19.37) [*n*/R] {*n*/R}	(140.2 ± 20.11) [*n*/R] {*n*/R}	RCI (*n*.S.)	0.27 [−0.34, 0.87] (*p* = 0.39)	-	-
	Johnson; 2008; USA	Number correct (max. 15) (NES2 Pattern Comparison Test)	*n*/R	*n*/R	(14.5 ± 0.9) [14.3 ± 1.8] {*n*/R}	(14.5 ± 0.7) [14.9 ± 0.3] {*n*/R}	Student’s paired *t*-test (*n*.S.)	0.00 [−0.86, 0.86] (*p* = 1.00)	−0.44 [−1.30, 0.43] (*p* = 0.33)	-
		Mean response time-correct (s) (NES2 Pattern Comparison Test)	*n*/R	*n*/R	(6.1 ± 2.3) [6.4 ± 2.3] {*n*/R}	(6.8 ± 3.0) [5.9 ± 2.3] {*n*/R}		−0.25 [−1.11, 0.61] (*p* = 0.33)	0.21 [−0.65, 1.07] (*p* = 0.63)	-
		Stroop test Mean RT, read words-black (ms)	*n*/R	*n*/R	(844 ± 239) [945 ± 185] {*n*/R}	(1040 ± 380) [891 ± 222] {*n*/R}		−0.6 [−1.48, 0.28] (*p* = 0.33)	0.25 [−0.61, 1.12] (*p* = 0.33)	-
		Stroop test Mean RT, read words-color (ms)	*n*/R	*n*/R	(753 ± 210) [883 ± 213] {*n*/R}	(788 ± 200) [804 ± 202] {*n*/R}		−0.16 [−1.02, 0.69] (*p* = 0.33)	0.36 [−0.50, 1.23] (*p* = 0.33)	-
		Stroop test Mean RT, name colors (ms)	*n*/R	*n*/R	(1008 ± 217) [1014 ± 193] {*n*/R}	(919 ± 173) [951 ± 220] {*n*/R}		0.43 [−0.44, 1.30] (*p* = 0.33)	0.29 [−0.57, 1.16] (*p* = 0.33)	-

Note: CANTAB: Cambridge Neuropsychological Test Automated Battery. WTAR: Wechsler Test of Adult Reading. CNSVS: CNS Vital Signs testing platform, GMLT: Groton Maze Learning Test. MOT: Motor screening task. AST: Attention switching task. VRM: Verbal recognition memory. PAL: Paired associated learning. VBM: Verbal memory test. VIM: Visual memory test. NVRT: Non-verbal reasoning test. SAT: Shifting attention test. FTT: Finger tapping test. SDC: Symbol-digit coding test. ST: Stroop test. CNSVS ver. CPT: Continuous performance task. rANOVA: Repeated measures analyses of variance. RCI: Reliable Change Index. ANOVA: analyses of variance. P: Placebo control group. A: Active intervention group. HA: High Astaxanthin group. LA: Low Astaxanthin group. P: Placebo group. *n*/R: Not reported. *n*.S.: Not significant (this abbreviation was used if a significantly low *p*-value was not shown in the paper).

**Table 4:** Quality assessment scores of included studies using modified Delphi list.

Lead Author; Year; Country	Q1	Q2	Q3	Q4	Q5	Q6	Q7	Q8	Q9	Q10	Q11	Q12	Q13	Q14	Total score (Max. = 14)
**Power; 2018; Ireland**	Y	Y	N	Y	Y	Y	Y	Y	?	Y	Y	Y	?	N	10
**Lindberg; 2018; USA**	Y	Y	?	Y	Y	Y	Y	?	?	Y	N	Y	Y	N	9
**Renzi-Hammond; 2017; USA**	Y	Y	?	Y	Y	Y	Y	?	N	Y	N	Y	Y	N	9
**Hammond; 2017; USA**	Y	Y	Y	Y	Y	Y	Y	?	N	Y	N	Y	Y	N	10
**Johnson; 2008; USA**	Y	Y	Y	Y	Y	Y	Y	Y	?	N	N	N	Y	N	9
**Katagiri; 2012; Japan**	Y	Y	Y	Y	Y	Y	Y	Y	N	N	Y	Y	Y	N	11
**Hayashi; 2018; Japan**	Y	?	Y	Y	Y	Y	Y	Y	N	N	Y	Y	Y	N	10
**Total score across studies**	7	6	4	7	7	7	7	4	0	4	3	6	6	0	–

Note: Q1: Random Allocation. Q2: Treatment Allocation concealed. Q3: Groups/Subjects similar at baseline regarding important prognostic values. Q4: Eligibility Criteria specified. Q5: Blinded outcome assessor. Q6: Care Provider blinded. Q7: Patient blinded. Q8: Point Estimates and Measures of Variability presented for the primary outcome measures. Q9. Intention-to-treat Analysis. Q10: details of Random Allocation methods. Q11: Adequate Description of the Control/Comparison group. Q12: Between-group statistical comparison. Q13: reporting Dropouts. Q14: reporting CONSORT statement. Y: Yes, the study met the criteria of the question. N: No; the study did not meet the criteria of the question? No information or the study was not the case with the question.

## Supplementary Materials

The following are available online at https://www.mdpi.com/2072-6643/12/3/617/s1, Table S1: PRISMA 2009 Checklist, Table S2: Search terms and strategy.
