# Supplementary material for: Effects of Lutein and Astaxanthin Intake on the Improvement of Cognitive Functions among Healthy Adults: A Systematic Review of Randomized Controlled Trials
Source: Nutrients. 2020 Feb 27;12(3):617. doi: 10.3390/nu12030617 (PMC7146131; doi:10.3390/nu12030617)
Supplement: Supplementary file 1 [file nutrients-12-00617-s001.zip › Supplementary Table2.pdf]

Search terms

PubMed & other databases (EMBASE, Web of Science, PsycINFO)

We use “Mesh” filter which can only use at PubMed in the below search terms, however, the search terms will be adapted for use with various bibliographic databases in combination with database specific filters, where available when we search other databases.

#1	"Cognition"[Mesh] OR "Cognition"[Title/Abstract] OR "Cognitive function"[Title/Abstract] OR "Cognitive Dysfunction"[Mesh] OR "Cognitive Dysfunction"[Title/Abstract] OR "Cognitive decline"[Title/Abstract] OR "Cognitive impairment"[Title/Abstract] OR "Mild cognitive impairment"[Title/Abstract] OR "Dementia"[Mesh] OR "Dementia"[Title/Abstract] OR "Alzheimer Disease"[Mesh] OR "Alzheimer"[Title/Abstract] OR "Alzheimer's"[Title/Abstract] OR "Memory"[Mesh] OR "Memory"[Title/Abstract] OR "Attention"[Mesh] OR "Attention"[Title/Abstract] OR "Executive Function"[Mesh] OR "Executive Function"[Title/Abstract] OR "Working Memory"[Title/Abstract] OR "processing speed"[Title/Abstract] OR "visuospatial"[Title/Abstract] OR "verbal fluency"[Title/Abstract] OR "word fluency"[Title/Abstract] OR "Learning"[Mesh] OR "Learning"[Title/Abstract] OR "Thinking"[Mesh] OR "Thinking"[Title/Abstract]
#2	"Carotenoids"[Mesh] OR "Carotenoids"[Title/Abstract] OR "Carotenoid"[Title/Abstract] OR "Xanthophylls"[Mesh] OR "Xanthophylls"[Title/Abstract] OR "Xanthophyll"[Title/Abstract] OR "Lutein"[Mesh] OR "Lutein"[Title/Abstract] OR "Zeaxanthins"[Mesh] OR "Zeaxanthins"[Title/Abstract] OR "Zeaxanthin"[Title/Abstract] OR "Cryptoxanthins"[Mesh] OR "Cryptoxanthins"[Title/Abstract] OR "Cryptoxanthin"[Title/Abstract] OR "beta Carotene"[Mesh] OR "beta Carotene"[Title/Abstract] OR "Vitamin A"[Mesh] OR "Vitamin A"[Title/Abstract] OR "Astaxanthin"[Title/Abstract]
#3	"intervention"[Title/Abstract] OR "randomized"[Title/Abstract] OR "randomised"[Title/Abstract] OR "RCT"[Title/Abstract] OR "placebo"[Title/Abstract] OR "clinical trial"[Title/Abstract]
#4	#1 AND #2 AND #3

Cochrane library

#1	MeSH descriptor: [Cognition] explode all trees
#2	MeSH descriptor: [Cognitive Dysfunction] explode all trees
#3	MeSH descriptor: [Alzheimer Disease] explode all trees
#4	MeSH descriptor: [Dementia] explode all trees
#5	MeSH descriptor: [Memory] explode all trees
#6	MeSH descriptor: [Attention] explode all trees
#7	MeSH descriptor: [Executive Function] explode all trees
#8	MeSH descriptor: [Learning] explode all trees
#9	MeSH descriptor: [Thinking] explode all trees
#10	("Cognition" OR "Cognitive function" OR "Cognitive Dysfunction" OR "Cognitive decline" OR "Cognitive impairment" OR "Mild cognitive impairment" OR "Dementia" OR "Alzheimer" OR "Alzheimer's" OR "Memory" OR "Attention" OR "Executive Function" OR "Working Memory" OR "processing speed" OR "visuospatial" OR "verbal fluency" OR "word fluency" OR "Learning" OR "Thinking"):ti,ab,kw
#11	MeSH descriptor: [Carotenoids] explode all trees
#12	MeSH descriptor: [Xanthophylls] explode all trees
#13	MeSH descriptor: [Lutein] explode all trees
#14	MeSH descriptor: [Zeaxanthins] explode all trees
#15	MeSH descriptor: [Cryptoxanthins] explode all trees
#16	MeSH descriptor: [beta Carotene] explode all trees
#17	MeSH descriptor: [Vitamin A] explode all trees
#18	MeSH descriptor: [Retinoids] explode all trees
#19	("Carotenoids" OR "Carotenoid" OR "Xanthophylls" OR "Xanthophyll" OR "Lutein" OR "Zeaxanthins" OR "Zeaxanthin" OR "Cryptoxanthins" OR "Cryptoxanthin" OR "beta Carotene" OR "Vitamin A" OR "Astaxanthins" OR "Astaxanthin"):ti,ab,kw
#20	("intervention" OR "randomized" OR "randomised" OR "RCT" OR "placebo" OR "clinical trial"):ti,ab,kw
#21	(#1 OR #2 OR #3 OR #4 OR #5 OR #6 OR #7 OR #8 OR #9 OR #10) AND (#11 OR #12 OR #13 OR #14 OR #15 OR #16 OR #17 OR #18 OR #19) AND #20
